# No modulation of postprandial metabolism by transcutaneous auricular vagus nerve stimulation: a cross-over study in 15 healthy men

**DOI:** 10.1038/s41598-020-77430-2

**Published:** 2020-11-24

**Authors:** Andreas Vosseler, Dongxing Zhao, Louise Fritsche, Rainer Lehmann, Konstantinos Kantartzis, Dana M. Small, Andreas Peter, Hans-Ulrich Häring, Andreas L. Birkenfeld, Andreas Fritsche, Robert Wagner, Hubert Preißl, Stephanie Kullmann, Martin Heni

**Affiliations:** 1 Department of Internal Medicine IV, Division of Diabetology, Endocrinology and Nephrology, University Hospital Tübingen, Otfried-Müller-Str. 10, 72076 Tübingen, Germany; 2 Institute for Diabetes Research and Metabolic Diseases of the Helmholtz Center Munich at the University of Tübingen, Tübingen, Germany; 3 German Center for Diabetes Research (DZD e.V.), Neuherberg, Germany; 4 Institute for Clinical Chemistry and Pathobiochemistry, Department for Diagnostic Laboratory Medicine, University Hospital Tübingen, Tübingen, Germany; 5 Modern Diet and Physiology Research Center, Department of Psychiatry, Yale University, New Haven, CT USA

**Keywords:** Homeostasis, Translational research, Endocrine system and metabolic diseases

## Abstract

Experimental evidence suggests a crucial role of the autonomic nervous system in whole body metabolism with major regulatory effects of the parasympathetic branch in postprandial adaptation. However, the relative contribution of this mechanism is still not fully clear in humans. We therefore compared the effects of transcutaneous auricular vagus nerve stimulation (taVNS, Cerbomed Nemos) with sham stimulation during an oral glucose tolerance test in a randomized, single-blind, cross-over design in 15 healthy lean men. Stimulation was performed for 150 min, 30 min before and during the entire oral glucose tolerance test with stimulation cycles of 30 s of on-phase and 30 s of off-phase and a 25 Hz impulse. Heart rate variability and plasma catecholamine levels were assessed as proxies of autonomic tone in the periphery. Neither analyzed heart rate variability parameters nor plasma catecholamine levels were significantly different between the two conditions. Plasma glucose, insulin sensitivity and insulin secretion were also comparable between conditions. Thus, the applied taVNS device or protocol was unable to achieve significant effects on autonomic innervation in peripheral organs. Accordingly, glucose metabolism remained unaltered. Therefore, alternative approaches are necessary to investigate the importance of the autonomic nervous system in postprandial human metabolism.

## Introduction

The autonomic nervous system modulates systemic metabolism through the innervation of peripheral organs^1^. This appears to be of special importance in the postprandial setting when rapid adaptations in various tissues are crucial for a healthy response to this metabolic challenge. While this is achieved predominantly via direct cellular action of postprandial factors like insulin, the autonomic nervous system appears to modulate and coordinate those effects^2–4^. More specifically, the parasympathetic nervous system is important for postprandial metabolism, as increased parasympathetic nerve activity leads to improved insulin sensitivity, insulin secretion and glucose tolerance^2, 5, 6^.

The major parasympathetic nerve, the vagus nerve innervates the pancreas, the hepatic portal system, and most of the gastrointestinal tract. Vagal efferents stimulate pre- and postprandial insulin secretion from the pancreas^1, 7^ as well as hepatic insulin sensitivity and insulin clearance^8^. As most of these results are derived from studies in rodents, the relative contribution of the autonomic nervous system for glucose metabolism in humans is still not fully understood.

Non-invasive transcutaneous auricular vagus nerve stimulation (taVNS) is an approach to stimulate the auricular branch of the vagus nerve through the outer ear in humans. TaVNS can potentially activate organs indirectly via vagal afferent or directly by activation of the vagal efferents from the ear to peripheral organs^9^.

TaVNS is successfully used for the therapy of drug-resistant epilepsy^10^.

TaVNS activates the vagal afferents and influences various brain functions^11^. Parasympathetic vagal afferent signals are integrated in the nucleus of the solitary tract and further ascend to hypothalamus^12, 13^ and striatum^14^. These brain regions also integrate and process central signals and in turn regulate the vagal efferent. Though, evidence about the effects of taVNS on the vagal efferent is sparse, taVNS may serve as an interesting non-invasive intervention that may influence vagal efferent activities.

A prior study suggested that taVNS over 15 min regulates cardiac branches of the vagal efferent. For example, heart rate variability (HRV), an indicator of the vagal efferent activity of the cardiac branches, was immediately changed by taVNS, in a direction that points to a shift from sympathetic to parasympathetic tone^15^. In line, Badran et al., reported decreased heart rate and attenuate heart rate rebound during taVNS compared to sham stimulation^16^.

Furthermore, 30 min of taVNS reduced gastric frequency, i.e. the rhythmic contractions of the stomach^13^.

In contrast, 14 min of taVNS did not modulate the autonomic tone to visceral organs up to 120 min post stimulation in a recent study^17^.

Thus, most previous studies indicated that taVNS affects vagal outflow to the periphery and might therefore be a useful tool to experimentally modulate autonomic regulations in the body.

We now aimed to study the effect of immediate modulation of parasympathetic tone by taVNS with Cerbomed NEMOS on whole-body metabolism during an oral glucose challenge and hypothesized improved insulin sensitivity and insulin secretion. Therefore, we designed a randomized, placebo-stimulation controlled, single blind, cross-over study to test effects of vagus nerve stimulation on systemic metabolism and energy expenditure.

## Results

**Figure 1:**
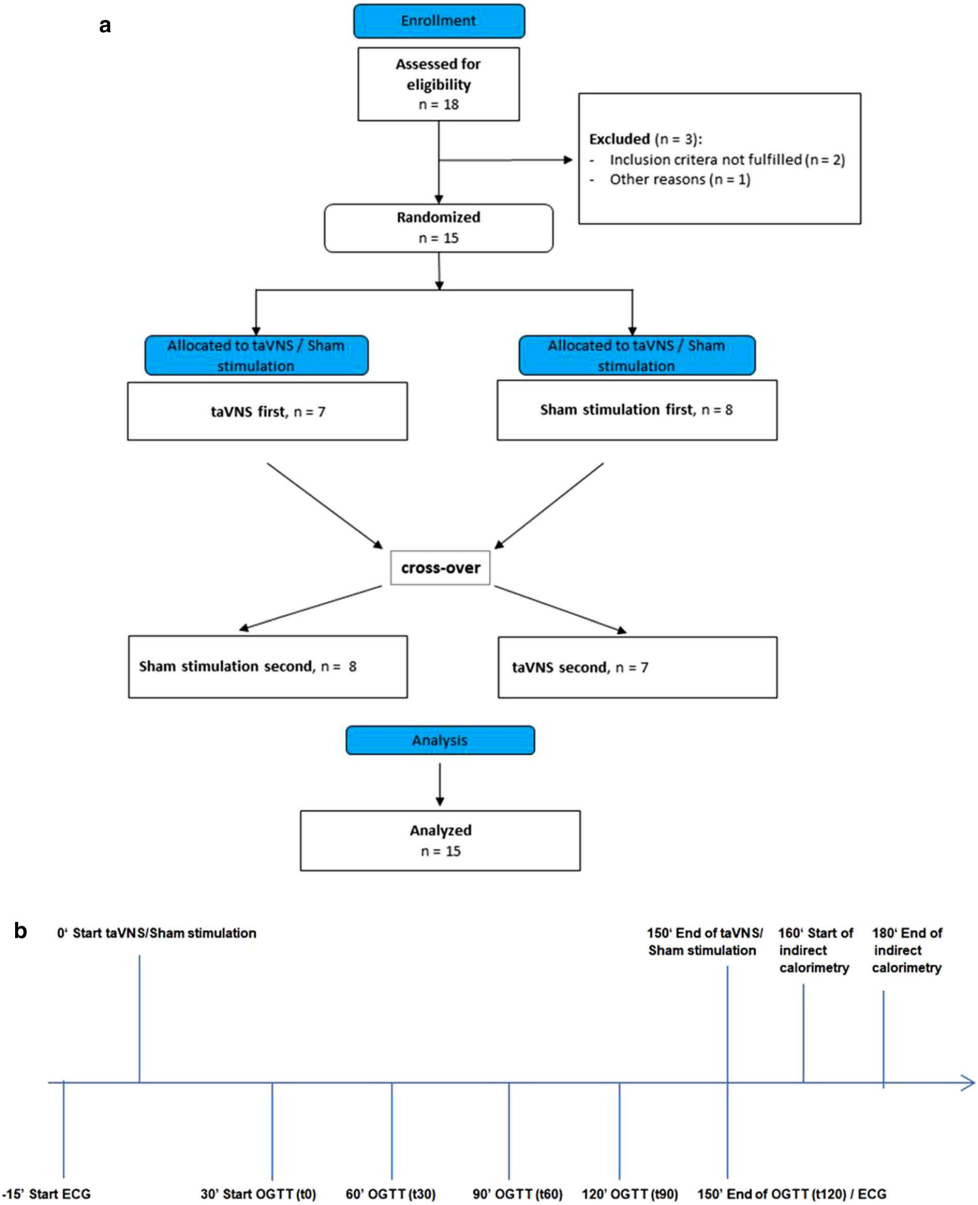
(**a**) Overview of the course of the study. (**b**) Schematic overview of the experiment. Electrocardiogram (ECG) was continuously recorded from timepoint -15 min before starting transcutaneous vagus nerve stimulation (taVNS) until the end of oral glucose tolerance test (OGTT) at 150 min. taVNS and sham stimulation were performed from timepoint 0 to 150 min. Start of OGTT was 30 min after initiation of taVNS/sham stimulation with intake of 75 g glucose. The last blood sample of OGTT was taken 2 h thereafter at timepoint 150 min. Indirect calorimetry was assessed after completion of the OGTT starting at 160 min.

**Figure 2:**
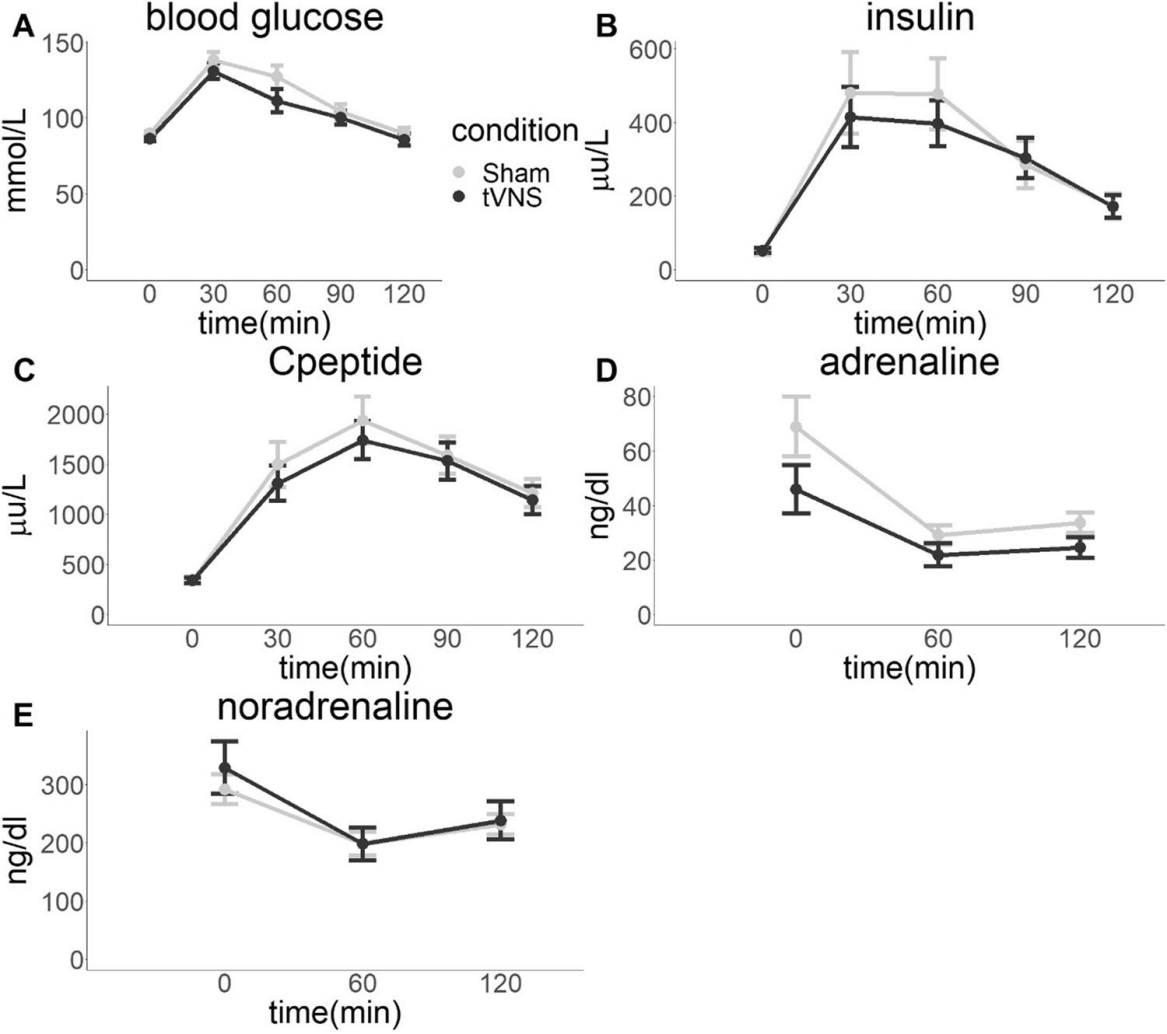
Endocrine and metabolic results. (**A**) Blood glucose, (**B**) serum insulin and (**C**) C-peptide increases during the oral glucose tolerance test (OGTT) that started at 0 min. Plasma adrenaline (**D**) and noradrenaline (**E**) decreased. However, transcutaneous vagus nerve stimulation (taVNS) did not significantly influence any of the hormone levels. Presented are means, error bars indicate standard errors.

The clinical characteristics of the fifteen healthy men who were included in the current study are presented in Table 1. In a cross-over design they received taVNS versus sham stimulation (Fig. 1). Endocrine and metabolic results are shown in Fig. 2. Detailed results of the linear mixed model analyses can be found in Table 2.

**Table 1:** Subject characteristics. Values are given as mean ± SD.

n	15
Age (years)	24 (± 3)
BMI (kg/m^2^)	22.9 (± 3.01)
Body fat content (%)	13.7 (± 2.9)
HbA1c (mmol/mol; %)	33.7 (± 2.6); 5.2 (± 0.2)
Waist-to-hip ratio	0.83 (± 0.04)

**Table 2:** Results of linear mixed model analysis on the effects of transcutaneous vagus nerve stimulation on heart rate variability and hormones.

Main effects	Degrees of freedom	F	*p*
**Mean heart rate**			
Time	4,56	14.60	< 0.0001
Condition	1,14	0.03	0.88
Time-by-condition interaction	4,55	1.47	0.22
**RMSSD**			
Time	4,56	1.76	0.15
Condition	1,14	0.80	0.39
Time-by-condition interaction	4,55	3.04	0.025
**LF/HF ratio**			
Time	4,56	9.69	< 0.001
Condition	1,14	0.03	0.86
Time-by-condition interaction	4,55	1.18	0.33
**Adrenaline**			
Time	1,41	2.05	0.16
Condition	1,41	1.72	0.20
Time-by-condition interaction	1,41	0.12	0.73
**Noradrenaline**			
Time	1,41	6.37	0.016
Condition	1,41	1.32	0.26
Time-by-condition interaction	1,41	0.04	0.84
**Blood glucose**			
Time	3,97	29.77	< 0.0001
Condition	1,97	0.85	0.36
Time-by-condition interaction	1,97	0.03	0.85
**Insulin**			
Time	3,97	26.83	< 0.0001
Condition	1,97	0.67	0.41
Time-by-condition interaction	1,97	0.39	0.76
**C-peptide**			
Time	3,97	41.72	< 0.0001
Condition	1,97	2.89	0.092
Time-by-condition interaction	1,97	0.59	0.44

Time point (min)	taVNS	Sham	
**Mean heart rate (bpm)**			
− 15	73.7 ± 2.7	74.2 ± 3.2	
0	66.8 ± 2.4	68.6 ± 2.5	
30	70.5 ± 2.3	72.3 ± 2.3	
60	73.4 ± 2.1	72.2 ± 2.4	
90	75.2 ± 2.1	73.4 ± 2.6	
120	73.5 ± 1.9	75.1 ± 2.4	
**RMSSD (ms)**			
− 15	33.3 ± 3.2	33.3 ± 4.1	
0	48.2 ± 5.0	39.8 ± 3.9	
30	45.9 ± 3.8	44.2 ± 4.6	
60	39.6 ± 3.0	39.6 ± 3.8	
90	37.2 ± 2.4	38.7 ± 3.3	
120	46.5 ± 4.7	36.6 ± 2.7	
**LF/HF ratio**			
− 15	2.6 ± 0.2	2.7 ± 0.3	
0	3.2 ± 0.4	2.9 ± 0.4	
30	2.8 ± 0.2	3.3 ± 0.4	
60	6.2 ± 0.9	5.4 ± 0.8	
90	4.6 ± 0.2	4.5 ± 0.5	
120	2.7 ± 0.2	3.1 ± 0.4	
**Adrenaline (ng/dL)**			
0	45.8 ± 8.9	68.9 ± 11.0	
60	21.8 ± 4.3	29.0 ± 3.6	
120	24.5 ± 3.7	33.7 ± 3.8	
**Noradrenaline (ng/dL)**			
0	329 ± 45	292 ± 25	
60	198 ± 28	198 ± 20	
120	238 ± 33	232 ± 17	
**Blood glucose (mg/dL)**			
0	86.4 ± 1.9	89.8 ± 1.6	
30	130.7 ± 5.2	138.3 ± 5.2	
60	111.3 ± 7.6	127.0 ± 7.6	
90	100.1 ± 4.6	104.1 ± 4.9	
120	85.7 ± 4.0	89.9 ± 3.4	
**Insulin (pmol/l)**			
0	51.9 ± 6.9	47.8 ± 8.2	
30	414.7 ± 82.4	480.6 ± 111.3	
60	397.7 ± 62.3	477.9 ± 96.8	
90	303.3 ± 55.0	285.7 ± 63.9	
120	171.3 ± 31.0	172.3 ± 34.6	
**C-peptide (pmol/l)**			
0	338.3 ± 29.4	338.7 ± 36.0	
30	1308.8 ± 176.9	1493.2 ± 226.1	
60	1737.3 ± 190.9	1937.5 ± 234.5	
90	1530.8 ± 186.1	1588.2 ± 185.3	
120	1139.3 ± 137.6	1208.7 ± 139.0	

*RMSSD* root mean square of successive differences; *LF/HF ratio*: low frequency to high frequency ratio.

### Effects of taVNS on peripheral vagal activity

Mean heart rate decreased after the oral glucose tolerance test (OGTT) (main effect of time, *p* < 0.0001). Though, mean heart rate did not differ between taVNS and sham stimulation (main effect of condition, *p* = 0.88), nor was there a time-by-condition interaction (*p* = 0.22).

Likewise, root mean square of successive differences (RMSSD) as an indicator for vagal tone did not change over time (main effect of time, *p* = 0.15). Moreover, RMSSD did not differ between taVNS and sham conditions (main effect of condition, *p* = 0.39). However, there was a significant time-by-condition interaction (*p* = 0.025), but post hoc contrasts did not show significant differences between conditions at any time point (all p_Holm_ > 0.1).

Low frequency to high frequency ratio (LF/HF) indicates sympathovagal balance, and higher LF/HF indicates dominance of sympathetic activity, and vice versa. We found that LF/HF increased after OGTT (main effect of time, *p* < 0.0001). However, LF/HF did not differ between conditions (main effect of condition, *p* = 0.86) and there was no time-by-condition interaction (*p* = 0.33).

As a second approach to address peripheral autonomic tone, we analyzed plasma catecholamines, the effectors of the sympathetic nervous system. Plasma noradrenaline levels decreased over time (*p* = 0.016). There was no significant time effect for adrenaline (*p* = 0.16). There was no statistically significant difference between taVNS and sham stimulation (adrenaline *p* = 0.20, noradrenaline *p* = 0.26) and no time-by-condition interaction was detected (adrenaline *p* = 0.73, noradrenaline *p* = 0.84).

### Effects of taVNS on whole-body glucose metabolism

Glucose excursions during the OGTT where comparable between taVNS and sham stimulation (AUC_glucose_ *p* = 0.1). Hence, glucose tolerance assessed as plasma glucose 2 h after initiation of OGTT, was not different between the two conditions (*p* = 0.4).

As readout for insulin secretion of the pancreatic beta cells, we analyzed serum insulin and C-peptide. In both conditions, there was no difference between serum insulin and C-peptide concentrations (main effect of condition, insulin *p* = 0.41, C-peptide *p* = 0.092).

Neither insulin sensitivity (ISI Matsuda *p* = 0.9; NEFA ISI *p* = 0.8) nor insulin secretion (Disposition index *p* = 0.2) was statistically significantly different between taVNS and sham condition.

### Effects of taVNS on resting energy expenditure and post-load substrate oxidation

Resting energy expenditure was comparable between conditions (*p* = 0.9). In the postprandial condition 2 h after OGTT, the preferential energy source was glucose in both conditions as indicated by an RQ around 1.0 (*p* = 0.5).

## Discussion

In the current study, we measured effects of a non-invasive vagus nerve stimulation on sympathetic and parasympathetic responses during an oral glucose tolerance test in healthy men. We found no significant influence of our stimulation approach on the tested parameters for autonomic balance. Accordingly, none of the analyzed glycemic traits were changed by the stimulation. Insulin sensitivity, insulin secretion, glucose tolerance and resting energy expenditure during the oral glucose tolerance tests did not differ between vagus stimulation and sham condition. TaVNS did not change sympathetic or parasympathetic tone to the heart as there were no detectable effects on heart rate variability. Finally, plasma catecholamines, the neurotransmitters of the sympathetic nervous system, were also unaffected by the stimulation. Thus, our data do not support major taVNS effects of taVNS on peripheral tissues.

However, taVNS is a well validated tool for vagal afferent stimulation that affects different brain functions^11, 15, 18–20^. These functions though were not able to modulate efferent outflows. Therefore, vagal afferent/efferent interaction appears to be unaffected by our current approach. How this interaction is regulated in detail is still not fully understood and should be further investigated in mechanistic studies at the molecular level.

One reason for the ineffectiveness of taVNS with the Nemos device could be that its vagal afferent stimulation in the auricular branch did not influence efferent activity towards the body.

Furthermore, an experiment in rodents suggested that a brain-gut communication occurs by directly stimulating the right vagus nerve^14^ whereas in our study stimulation electrodes were placed in the left ear due to safety concerns when stimulating the right ear in humans.

The stimulation electrode can be applicated in different regions at the outer ear (tragus, concha or cymba concha), however, it is not fully known which region and which ear is the most reliable for taVNS as the innervation of the auricular branch of the vagus nerve is still not fully clear^21^.

As the optimal stimulation frequency, intensity and duration for the intended effects are unknown, we might not have picked optimal parameters. Furthermore, potential technical issues as well as electrode size and fit could have limited potential effects.

An increased stimulation duration could possibly show an effect of taVNS on glucose metabolism as Huang et al. showed positive effects of taVNS on glucose metabolism during a period of 12 weeks in persons with metabolic syndrome^22^.

Clancy et al. reported increased heart rate variability in response to taVNS, indicating a shift from sympathetic to parasympathetic predominance in a much larger cohort compared to ours^15^. In addition, the stimulation protocol and trial setting of our current study was different from Clancy et al., who performed taVNS with another device, different stimulation protocol and included female and male participants whereas in our study only male persons were included. Their device stimulated for 15 min continuously^15^, whereas we used a repetitive sequence with 30 s of stimulation followed by 30 s pause for a longer period of time. Furthermore, no metabolic challenge was applied in the study of Clancy et al.^15^.

In contrast to Clancy’s positive results, Borges et al. could not detect any difference between transcutaneous auricular vagus nerve stimulation and sham stimulation on cardiac vagal activity in 61 healthy men^23^.

Thus, possible slight effects of the stimulation might have been masked by stronger effects of the metabolic alterations in our study.

Although vagus stimulation did not affect plasma catecholamine courses, there was a time effect of adrenaline and noradrenaline in both conditions. Adrenaline and noradrenaline levels were higher at time point 0 and declined during the OGTT. This could reflect the well-known shift from sympathetic towards parasympathetic tone in the postprandial state^1, 24, 25^. However, as we did not study catecholamines without glucose intake, we cannot exclude other potential contributors to this response.

In conclusion, auricular vagus nerve stimulation with the Nemos device had no major acute effects on the autonomic regulation of peripheral organs and did therefore neither alter insulin sensitivity, insulin secretion, resting energy expenditure nor sympathovagal balance. The physiological significance of the autonomic nervous system for glucose metabolism in humans must therefore be investigated with alternative approaches, e.g. either by biofeedback paradigmes or pharmacologically.

## Methods

Fifteen male healthy volunteers at an age between 18 and 31 years were included (further details are provided in Table 1). Body mass index (BMI) was between 19.3 and 25.2 kg/m^2^, body fat content was between 9.5 and 22.5% as measured by bioelectrical impedance testing (BIA 101 by Akern Srl, Florence, Italy) and estimated with Cyprus 2.7 Body Composition Analysis Software (RJL Systems, Michigan, USA). Subject characteristics are shown in Table 1.

The sample size (n = 15) provides 80% power to detect effect size f = 0.35 when setting the alpha-level to 0.05 (calculated with Gpower 3.1).

The study protocol was approved by the ethics committee of the medical faculty of the University Tübingen. Written informed consent was obtained from all study participants and all research was performed in accordance with relevant guidelines/regulations. The study was pre-registered at clinicaltrials.gov (NCT03615209; 03/08/2018).

An overview of the course of the study is given in Fig. 1.

Non-invasive transcutaneous vagal stimulation was applied with the Cerbomed NEMOS device, an earpiece with titanium electrodes that is placed in the cymba conchae of the left external ear and in upside down position (ear lobe) for sham stimulation . The device stimulates the auricular branch of the nervus vagus with a mild electrical current with stimulation cycles of 30 s of on-phase and 30 s of off-phase and a 25 Hz impulse.

Auricular vagus nerve stimulation (and sham stimulation, respectively) was performed 30 min before and during the entire OGTT (150 min overall) in randomized cross-over design in the morning of two different days with 5 to 16 days washout period in a randomized single-blind design.

Stimulation procedure was done according to the protocol of Frangos et al.^11^. Stimulus intensity was adjusted by the participants from 0.1 mA in 0.1 mA increments until the person reported a “tingling” sensation, but no pain^11^. Stimulation intensity was 2.5 mA ± SD 0.9 in taVNS and 3.2 mA ± SD 1.5 in sham condition.

All persons underwent an oral glucose tolerance test. After an overnight fast, participants ingested a solution containing 75 g glucose over 5 min (Accu-Chek Dextrose OGT, Roche). Before, and 30, 60, 90 and 120 min after glucose ingestion, blood samples were obtained following standard procedures^26^. Oral glucose tolerance test was performed to address dynamic insulin secretion from the pancreatic beta cells and insulin sensitivity from plasma glucose, C-peptide and insulin responses after ingestion of glucose solution. OGTT is a frequently used tool to assess insulin secretion and insulin sensitivity and can furthermore assess glucose tolerance^27^.

HbA1c was tested at baseline, serum insulin levels, C-peptide, plasma glucose and free fatty acids were measured at all five timepoints. All measurements were performed in a routine diagnostic laboratory that is accredited with the German accredited body (DAkkS). Glucose values were measured directly using a bedside glucose analyzer (Biosen C-line, EKF-diagnostic GmbH, Barleben, Germany). Serum insulin and C-peptide levels were determined by an immunoassay with ADVIA Centaur XP Immunoassay System (Siemens Healthineers, Eschborn, Germany).

Plasma concentrations of total non-esterified fatty acids (NEFA) were measured with an enzymatic method (WAKO Chemicals, Neuss, Germany). HbA1c measurements were performed using Tosoh glycohemoglobin analyzer HLC-723G8 (Tosoh Bioscience, Tokyo, Japan).

Insulin sensitivity and insulin secretion was calculated from the OGTT as described previously^28^.

Plasma catecholamines adrenaline and noradrenaline were measured at baseline and 60 and 120 min after glucose ingestion. Catecholamines were analyzed with high performance liquid chromatography (HPLC) using a commercial kit (Kits No 5000, Chromsystems, Grafelfingen, Germany). Adrenaline and noradrenaline were isolated from plasma prior to chromatographic separation by solid phase extraction. After sample pre-treatment, an isocratic HPLC analysis was performed with a flow rate of 1 ml/min and a total run time of 20 min. The limit of detection (LOD) for adrenaline is 3 ng/dl, and for noradrenaline 10 ng/dl.

Electrocardiogram (ECG) recordings were performed to analyze heart rate variability as a surrogate parameter for autonomic nerve activation and parasympathetic projections to the heart. ECG was continuously recorded 15 min before starting the electric stimulation until the end of OGTT. HRV analysis was done in 10 min intervals in resting state, before the start of the stimulation and 5 min before blood extraction. ECG was recorded with Biopac MP 36 (Biopac Systems, Inc., Goleta, CA) and analyzed with Matlab (Mathworks, Inc. USA).

Standard ECG electrodes were attached to the chest wall. ECG was recorded at 2.5 kHz and transduced, amplified and filtered with a low pass filter at 25 Hz and a high pass filter at 1 Hz. The data were visually inspected for artifact correction in Artiifact^29^. Data with less than 2 min continuous measurement (uninterrupted by movement artifacts) were excluded. Inter-beat intervals calculated from visually inspected data were then processed in Artiifact to correct for ectopic peaks. Root mean square of successive differences (RMSSD) in inter-beat intervals was calculated in the time domain as an indicator of the vagal tone. High-frequency (0.15–0.50 Hz, HF) component and low-frequency (0.05–0.14 Hz, LF) was calculated in the frequency domain, and the low-frequency (LF/HF) ratio was calculated as an indicator of the vagal tone^30, 31^.

ECG was recorded for 5 min at baseline and every 15 min after glucose intake. HRV parameters collected for each 5 min time bins were then analyzed in mixed effect models.

Resting energy expenditure was assessed after completion of the OGTT and the taVNS stimulation. Energy expenditure after vagus nerve and sham stimulation was calculated by indirect calorimetry measurements with Vyntus CPX (Vyaire Medical, Illinois, USA). O_2_ consumption and CO_2_ production was measured for 15 min. To correct for monitor-specific deviations and eliminate the influence of inherent variability of the device on the measurement results, individual calibration control evaluation (ICcE) was applied^32^.

Statistical analysis was performed using SAS 9.4 (SAS Institute, Cary, NC). All results are presented as mean ± SD. *p* < 0.05 was considered statistically significant.

Measurements for major outcomes were subtracted from baseline measurements and boxcox-transformed if needed to fulfill the assumption of normally distributed residuals. Mixed effect models were performed on that data, with main effects of time, taVNS, and their interaction effects as fixed effects, the subjects with random intercepts, and the visit order as a dummy variable. Denominator degrees of freedom were estimated using the default method in SAS, which is a containment method. The variance–covariance structure providing the best fit was chosen based on the minimum value of Akaike’s Information Criterion (AIC). Significant interaction effects were followed by post hoc contrasts at each time point between conditions, with stepdown Bonferroni (Holm) correction for multiple testing. Metabolic results and the resting energy expenditure and respiratory quotient were analyzed using paired *t* tests between conditions.

## Data Availability

The data are not publicly available due to them containing information that could compromise research participant privacy/consent.
